# Unfolding the Effects of Acute Cardiovascular Exercise on Neural Correlates of Motor Learning Using Convolutional Neural Networks

**DOI:** 10.3389/fnins.2019.01215

**Published:** 2019-11-14

**Authors:** Arna Ghosh, Fabien Dal Maso, Marc Roig, Georgios D. Mitsis, Marie-Hélène Boudrias

**Affiliations:** ^1^Integrated Program in Neuroscience, McGill University, Montreal, QC, Canada; ^2^École de Kinéiologie et des Sciences de l'Activité Physique, Université de Montréal, Montreal, QC, Canada; ^3^School of Physical and Occupational Therapy, McGill University, Montreal, QC, Canada; ^4^Center for Interdisciplinary Research in Rehabilitation of Greater Montreal (CRIR), Montreal, QC, Canada; ^5^Department of Bioengineering, McGill University, Montreal, QC, Canada

**Keywords:** motor learning, convolutional neural network (CNN), cardiovascular exercise, deep learning, EEG

## Abstract

Cardiovascular exercise is known to promote the consolidation of newly acquired motor skills. Previous studies seeking to understand the neural correlates underlying motor memory consolidation that is modulated by exercise, have relied so far on using traditional statistical approaches for *a priori* selected features from neuroimaging data, including EEG. With recent advances in machine learning, data-driven techniques such as deep learning have shown great potential for EEG data decoding for brain-computer interfaces, but have not been explored in the context of exercise. Here, we present a novel Convolutional Neural Network (CNN)-based pipeline for analysis of EEG data to study the brain areas and spectral EEG measures modulated by exercise. To the best of our knowledge, this work is the first one to demonstrate the ability of CNNs to be trained in a limited sample size setting. Our approach revealed discriminative spectral features within a refined frequency band (27–29 Hz) as compared to the wider beta bandwidth (15–30 Hz), which is commonly used in data analyses, as well as corresponding brain regions that were modulated by exercise. These results indicate the presence of finer EEG spectral features that could have been overlooked using conventional hypothesis-driven statistical approaches. Our study thus demonstrates the feasibility of using deep network architectures for neuroimaging analysis, even in small-scale studies, to identify robust brain biomarkers and investigate neuroscience-based questions.

## 1. Introduction

A single bout of cardiovascular exercise, when performed in close temporal proximity to a session of visuomotor skill practice has been shown to facilitate motor memory consolidation (Roig et al., 2013; Dal Maso et al., 2018). The positive effects of exercise on motor memory have been associated with a variety of events at the molecular and systems level. These involve an increased concentration of neurotrophin molecules such as brain-derived neurotrophic factors (BDNF), vascular endothelial growth factor (VEGF) and insulin-like growth factor (IGF-1). In turn, these factors mediate downstream effects like neurogenesis and synaptogenesis, which form the basis of events underlying neuroplasticity. Increased corticospinal excitability has also been observed during the memory consolidation period and is thought to facilitate synaptic transmission between neuronal networks involved in motor skill practice (Ostadan et al., 2016). However, the precise contribution of distinct brain areas and networks associated with the positive effects of exercise on motor memory consolidation remain largely unknown. Understanding how the brain is altered by exercise could hold the key to designing therapies that could optimize the neurophysiological changes associated with motor memory consolidation for varied purposes (Hendricks et al., 2002).

Electroencephalography (EEG) is a technique used to study the electrical activity originating in different brain areas. The EEG signal arises from synchronized postsynaptic potentials of neurons that generate electrophysiological oscillations in different frequency bands. During movement, the EEG signal power spectrum within the alpha (8–12 Hz) and beta (15–29 Hz) range decreases in amplitude and this is thought to reflect increased excitability of neurons in the sensorimotor areas (Salmelin et al., 1995; Crone et al., 1998; Neuper and Pfurtscheller, 2001; Pfurtscheller et al., 2003). This phenomenon is termed Event-Related Desynchronization (ERD). Various studies have reported that alpha- and beta-band ERD patterns are modulated during motor skill learning (Zhuang et al., 1997; Boonstra et al., 2007; Houweling et al., 2008). There is also converging evidence toward an association between cortical oscillations in the motor cortex and neuroplasticity events underlying motor memory consolidation (Boonstra et al., 2007; Pollok et al., 2014). Using EEG, we recently described the oscillatory patterns in brain electrical activity while subjects performed a handgrip task (Dal Maso et al., 2018). Using a time-frequency decomposition-based analysis pipeline, we found a significant decrease in post-exercise beta-band ERD in EEG electrodes located over the sensorimotor area in both hemispheres. Additionally, changes in beta-band ERD were associated with better skill retention 24 h after motor practice. These results suggest that changes in brain oscillatory patterns occur when motor learning is combined with acute exercise and that some of these changes have implications for skill retention. However, as these inferences were drawn from a hypothesis-driven approach, whereby standard EEG frequency bands from pre-selected electrodes were considered, the existence of more subtle, fine-scale neurophysiological features that are modulated by a single bout of exercise cannot be excluded.

Neural network models, particularly deep learning (DL) models, have been successful in identifying optimal discriminative features in a given dataset (LeCun et al., 2015). Convolutional Neural Networks (CNN) and Recurrent Neural Networks (RNN) have been applied to computer vision and speech processing datasets (Krizhevsky et al., 2012; Graves et al., 2013; Karpathy et al., 2014; Zhang and LeCun, 2015) with great success. These approaches have also been used in the context of neuroimaging to identify features for structural and functional magnetic resonance imaging data analysis (Plis et al., 2014) and EEG data decoding (Bashivan et al., 2015; Thodoroff et al., 2016; Schirrmeister et al., 2017b) among others.

CNNs are artificial neural networks that can learn low-level patterns in a given dataset by using convolution operations as a key component. CNN architectures may range from shallow architectures with just one convolutional layer (Abdel-Hamid et al., 2014), deep CNNs with multiple sequential convolutional layers (Krizhevsky et al., 2012) to very deep architectures with over 1,000 layers (He et al., 2015). CNNs have an edge over other machine learning models as they are well suited for end-to-end learning, i.e., learning from raw data without any *a priori* feature selection and they can exploit hierarchical structures that may be present in the data.

Although the field of EEG signal decoding has recently seen a surge of papers (Tjepkema-Cloostermans et al., 2018; Yannick et al., 2019; Yuan et al., 2019) involving the use of DL, the applicability of DL models has been primarily restricted to the classification of EEG data segments into known categories. The usefulness of CNNs to improve our understanding of the neural substrates underlying observed behaviors is less straightforward, primarily due to the difficulty associated with the visualization and interpretation of the feature space learned by DL architectures, e.g., CNNs. For instance, Schirrmeister et al. (2017b) proposed a systematic CNN framework for EEG decoding, including the impact of various architectural considerations on decoding performance. However, they presented a feature validation approach to understand which *a priori* selected features were used by the CNN rather than a feature discovery approach.

In this context, the aim of the present study was to develop a data-driven approach for studying the positive effects of exercise on motor learning by investigating the EEG-based ERD patterns during an isometric motor grip execution in healthy young subjects. We aimed to identify specific EEG spectral features modulated by exercise and further investigate if these features were related to skill retention performance. The subjects performed a repetition of isometric handgrips before and after a session of intense cycling exercise (exercise group) or rest for the same period (control group). In addition to isometric handgrips, subjects also practiced a new motor tracking task with their dominant hand in close proximity to the exercise or rest session. To identify the neurophysiological substrates underlying the positive effects of exercise, we used a CNN-based deep network architecture to identify exercise-induced changes in neural activity from EEG signals recorded during the handgrip task. Since neural networks are known to be universal function approximators (Hornik et al., 1989), with the capability of identifying linear as well as non-linear boundaries in high-dimensional data spaces, this allowed us to differentiate the exercise and control groups in the EEG time-frequency data space. The training was carried out in a hierarchical structure—initially in the time-frequency domain and subsequently for topographical maps of ERD pattern. Visualizing the features after each stage of training allowed us to identify frequency bands as well as the corresponding brain regions modulated by the positive effects of acute exercise on motor learning. Moreover, the majority of previous related DL studies used datasets comprising of hundreds of subjects for training purposes (Plis et al., 2014; Schirrmeister et al., 2017a). Therefore, one of our main goals was to develop a DL-based method that is suitable for neuroimaging studies with smaller subject numbers, which is frequently the case. To this end, we added a regularizer (adversary component) to the CNN, which prevented the latter from learning subject-specific features, thus favoring the identification of group-specific features. The proposed approach revealed that the CNN-extracted features were strongly correlated to the improvement in motor learning scores. Visualizing these features revealed finer frequency bands and corresponding brain regions where ERD patterns were modulated by exercise. Therefore, the proposed analysis provided observational evidence for the identified frequency band-related ERD to be associated with the positive effects of exercise on motor memory consolidation.

## 2. Dataset

The experiment and data collection are described in detail in Dal Maso et al. (2018). Briefly, 25 right-handed healthy subjects were recruited and assigned to the Control (CON, *n* = 13 subjects) or Exercise (EXE, *n* = 12 subjects) groups in matched blocks. The blocks of subjects were created with similar age, gender, body mass index, working and episodic memory as well as cardiorespiratory fitness. All subjects signed a written consent form according to the research protocol that complied with the recommendations of the declaration of Helsinki for investigation of human participants and was approved by our local ethics committee (CRIR-1134-0116).

Each subject reported to the laboratory on four occasions as described in Dal Maso et al. (2018). Visit 1 required the participants to go through a Graded Exercise Test (GXT), which was used to determine their cardiorespiratory fitness. Visit 2 was conducted at least 48 h after the GXT to avoid potential long-term effects of exercise on memory (Berchtold et al., 2005; Hopkins et al., 2012). EEG recordings were collected at baseline (before the exercise session) while subjects performed repetitions of visually cued isometric handgrips with their dominant right hand using a hand clench dynamometer (Biopac, Goleta, CA, USA). Each contraction was maintained for 3.5 s at 15% of each participant's maximum voluntary contraction (MVC). This was followed by a 3–5 s rest period. The baseline assessment was followed by the practice of a visuomotor tracking task (skill acquisition), which was used to calculate the motor learning score of each subject. Following the training period, participants were randomly assigned to two groups. The EXE group performed a bout of high-intensity interval cycling of 15 min, while the CON group rested on the cycle ergometer for the same amount of time. EEG recordings similar to baseline were repeated 30, 60, and 90 min after the exercise or rest period. During visits 3 and 4, two blocks of the visuomotor tracking task were performed 8 and 24 h after the exercise or rest period.

EEG activity was recorded using a 64-channel ActiCap cap (BrainVision, Munich, Germany) with electrode locations arranged according to the 10–20 international system. The electrical conductive gel was inserted at each electrode site to keep impedances below 5 kΩ. EEG signals were referenced to the FCz electrode and sampled at 2,500 Hz.

## 3. Methods

The analysis pipeline was first applied to the time and frequency domain data without incorporating spatial information. Subsequently, it was applied to the data obtained by creating topographical maps corresponding to the distribution of activity within specific frequency bands across the cortex. The entire pipeline consisted of 3 segments, i.e., Preprocessing, CNN training, and cue-combination for Class Activation Map (ccCAM) generation.

### 3.1. Time-Frequency (TF) Maps

#### 3.1.1. Preprocessing

**Figure 1:**
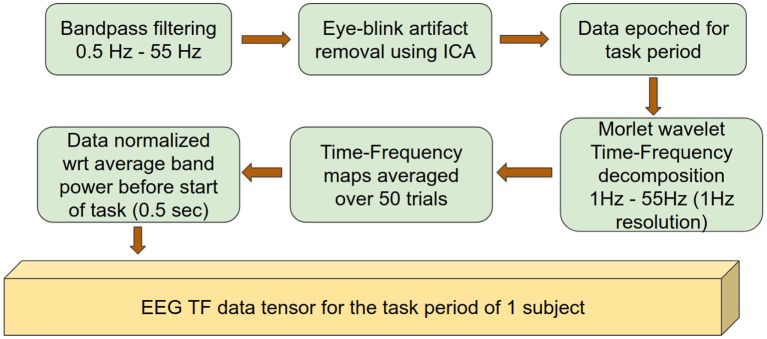
Data preprocessing pipeline outlining the preparation of data to be used as input to the deep network.

EEG data preprocessing was similar to that described previously (Dal Maso et al., 2018) and was performed using the Brainstorm Matlab toolbox (Tadel et al., 2011). The preprocessing pipeline is summarized in Figure 1. Briefly, EEG signals were bandpass-filtered between 0.5 and 55 Hz and average-referenced. The data were visually inspected and signal segments with artifacts were rejected. Independent component analysis (ICA) was subsequently applied (total number of components: 20) and eye-blink related components were rejected based on their topography and time signatures (Delorme and Makeig, 2004). The resulting data were epoched with respect to the period of time (3.5 s) corresponding to the appearance of the visual cue that triggered the initiation of the isometric handgrips (*n* = 50/subject). Finally, each epoch was visually inspected and those containing artifacts were manually removed. EEG electrodes with atypical power spectrum density were interpolated using spherical splines.

Morlet wavelet (wave number = 7) coefficients between 1 and 55 Hz with 1 Hz resolution were extracted to obtain time-frequency decompositions of the EEG data (Tallon-Baudry et al., 1996). The time-frequency data for each electrode were normalized with respect to their spectral power before the start of the grip event, using a window of 0.5 s. An average over all trials was then calculated in order to obtain a single time-frequency map for each electrode. Subsequent analysis was performed on the EEG recording segment corresponding to 0.5–3.5 s after the appearance of the visual cue, i.e., during the handgrip task. Finally, one time-frequency map was obtained for each electrode for each session (baseline, 30 min, 60 min or 90 min after exercise) and for each subject.

#### 3.1.2. CNN Training

**Figure 2:**
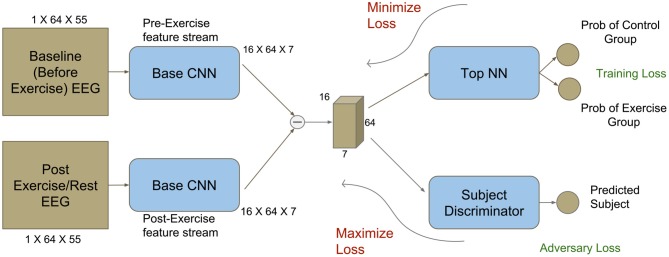
Modified deep network architecture with an adversary component (bottom right). The adversary component makes it feasible to use CNNs for identifying subject-invariant features in neuroimaging studies with a limited number of subjects. Dimensions corresponding to the obtained TF maps are also shown.

The proposed CNN architecture is shown in Figure 2. The preprocessed data for each session was rearranged to form 2D matrices comprising of the frequency spectra for all electrodes at a given time instant t. Each matrix had a dimension of 64 × 55 (64 electrodes × 55 frequency bands). For training the network, a pair of matrices was used—the first corresponding to time point t from the baseline session and the second corresponding to the same time point t from the post-intervention session. Each pair was labeled based on the respective group allocation (EXE or CON). Structuring the data in this fashion allowed the network to take into account the inter-subject variability in baseline measures and therefore did not require the experimenter to adopt techniques for normalizing the EEG signal from the post-intervention session with respect to the baseline session. Thus, the network was expected to capture the EEG features that were modulated by the effects of acute exercise.

##### 3.1.2.1. Dataset notation

***B*** and ***A*** represent the entire data tensor at baseline and post-intervention, respectively. Each data tensor consists of data matrices from all 25 subjects and timepoints. For subject *s*, the goal was to classify whether the tuple containing the matrices $B_{t}^{s}$ and $A_{t}^{s}$ (where *t* denotes timepoint) belongs to the EXE or CON groups. Matrices $B_{t}^{s}$ and $A_{t}^{s}$ were arranged so that they belonged to the set *R*^64*X*55^, where 64 is the number of electrodes and 55 is the number of frequency bands.

To identify EEG features modulated by exercise, we used a deep CNN that was trained to discriminate between EEG data from EXE and CON group. The network architecture is similar to the one described in Agrawal et al. (2015). Features from matrices $B_{t}^{s}$ and $A_{t}^{s}$ were extracted using a network termed Base CNN. The difference between the obtained feature vectors was passed to a discriminator network, termed Top NN, to predict the correct group to which each pair belonged to. The schematic view of the architecture is shown in Figure 2 and the details for each network's architecture are provided in Tables S1, S2, respectively. Using a sampling frequency of 2,500 Hz and a time period of interest of 3 s duration, each tuple input to the CNN was of the form ($B_{t}^{s}$,$A_{t}^{s}$) where timepoint *t* ∈ [1,7500] and subject *s* ∈ [1,25].

The convolutions performed in the Base CNN were with respect to the frequency direction and not the electrode (sensor) dimension. This is done because the frequency dimension was by definition arranged in terms of increasing frequencies, as opposed to the electrode dimension, which was not arranged in terms of the spatial locations of the electrodes in a meaningful manner. Consequently, the features extracted by the Base CNN corresponded to the frequency bands significantly affected by exercise. Therefore, all convolutional filters in the Base CNN were implemented as 1 × *n* 2D filters, where *n* is the extent of the filter in the frequency domain. The same holds for the Max-Pooling layers.

Initially, a network that did not include an adversary loss component (Figure S1A) was used; however, it was found that this network was able to learn subject-specific features as opposed to subject-invariant, exercise-related features. This is illustrated in Figure S2 and **Table 2**. In most neuroimaging studies examining the effect of exercise, the number of participants scanned was relatively low (Gutmann et al., 2015; Robertson and Marino, 2015; Huang et al., 2017), which typically prevents deep networks from learning subject-invariant features. To address this issue, we followed a domain adaptation approach. Specifically, each subject was considered as a separate domain comprising of subject-specific features along with subject-invariant, exercise-related features. Since our goal was to learn features mainly related to the effect of exercise on the consolidation of motor memory, we incorporated the domain confusion strategy (Tzeng et al., 2015) to train the network, thus adding the subject discriminator as an adversary (Figure 2, bottom right). Specifically, we included this network in parallel to the Top NN with similar model capacity (see Table S3 for architecture details).

##### 3.1.2.2. Network architecture notation

The feature extractor operation and parameters of the Base CNN are denoted as *f*_θ_*f*__ and θ_*f*_, respectively, the Top NN feature discrimination operator and its parameters by *h*_θ_*t*__ and θ_*t*_, respectively, while the subject discrimination operator and its parameters by *h*_θ_*s*__ and θ_*s*_, respectively. The input tuple is denoted by *x* and its corresponding group and subject labels by *y*_*g*_ and *y*_*s*_, respectively. We used the Negative Log Likelihood (NLL) loss for each classifier with the Adam optimizer (Kingma and Ba, 2014) in Torch (Collobert et al., 2011) for training the network. The Subject Discriminator was trained to minimize the subject prediction loss given by

(1) $$\begin{array}{l} J_{s}\left(\theta_{s},\theta_{f}\right)=-\left[\overset{m}{\underset{i=1}{\sum }}logh_{\theta_{s}}^{\left(y_{s}^{\left(i\right)}\right)}\left(f_{\theta_{f}}\left(x^{\left(i\right)}\right)\right)\right] \end{array}$$

The Top NN was trained to minimize the group prediction loss given by

(2) $$\begin{array}{l} J_{g}\left(\theta_{t},\theta_{f}\right)=-\left[\overset{m}{\underset{i=1}{\sum }}logh_{\theta_{t}}^{\left(y_{g}^{\left(i\right)}\right)}\left(f_{\theta_{f}}\left(x^{\left(i\right)}\right)\right)\right] \end{array}$$

We trained the feature extractor (Base CNN) to extract features that would be agnostic to the originating subject; therefore, the target distribution for the subject prediction network had a uniform distribution. Hence, we used the domain confusion loss (Tzeng et al., 2015) over the gradient reversal layer (Ganin et al., 2016). We also used the Kullback-Leibler (KL) divergence from the uniform distribution over 25 classes (25 subjects) as our loss metric. Conclusively, the Base CNN was trained to minimize the loss given by

(3) $$\begin{array}{l} J_{f}(\theta_{f},\theta_{t},\theta_{s})=-[\sum_{i=1}^{m}logh_{\theta_{t}}^{\left(y_{g}^{\left(i\right)}\right)}(f_{\theta_{f}}(x^{\left(i\right)}))]\\ \;+\lambda [\sum_{i=1}^{m}KL(U,h_{\theta_{s}}(f_{\theta_{f}}(x^{\left(i\right)}))] \end{array}$$

where *KL*(*P, Q*) denotes the KL divergence between distributions *P* & *Q*, *U* denotes the uniform distribution, *m* denotes the total number of training examples, and λ is a hyperparameter that determines the weight for the subject discrimination regularizer. Here, we used a 80-20 split of the data set, whereby 80% was used for training and 20% was used for validation.

For our experiments, we used two different cross-validation strategies (1) a train-validation split at the timepoint level and (2) a train-validation split at the subject level. In the timepoint level split strategy, 80% of all the data from all timepoints (aggregated from all subjects) were used for training the network. Therefore, the network was exposed to data from all subjects during training. This training strategy was used to observe the network's behavior during training, the effect of the subject adversary and further investigate the features extracted by the network. Contrastingly, the subject level split strategy used data from 23 subjects for training the network and the held-out 2 subjects were used to evaluate the network's performance on unseen or “novel” subjects. This strategy was used to assess the network's generalizability across subjects and compare it to other EEG decoding methods (10-fold cross-validation accuracy was compared across methods in Table 1). In addition, we added a random label-shuffling analysis within the subject level split strategy to check for selection bias in the results of our proposed methods.

**Table 1:** Comparison of CNN performance with alternative machine learning methods.

**Method**	**Average validation accuracy over 10 folds (%)**
Random forests	59.97 ± 5.21
Random forests with frequency bands	60.14 ± 5.59
Random forests with common spatial patterns	43.23 ± 4.44
CNN without baseline normalization architecture	56.59 ± 4.04
CNN without subject adversary	59.29 ± 4.61
**CNN with subject adversary (proposed)**	**74.85 ± 5.65**
Proposed CNN with labels shuffled at subject level	62.57 ± 3.92

*Bold values highlight the method with the best performance on the validation set*.

#### 3.1.3. ccCAM

An innovative contribution of the present work is the development of a novel method for the visualization of the features that guide the proposed network's decision. Although well-known techniques used in the computer vision literature include Global Average Pooling (GAP) (Zhou et al., 2016) and grad-CAM (Selvaraju et al., 2016), they are not well suited to the neurophysiological paradigm considered here. For instance, GAP requires averaging the activations of each filter map, i.e., each channel of the extracted feature tensor. This leads to loss of information related to electrode positions, as convolutions were performed only in the frequency domain. Consequently, we were unable to obtain adequate classification accuracy (≈56%) with a GAP layer in the network. Grad-CAM is sensitive to the absolute scale of variability in the features in the input data and, as a result, it yielded results that were biased toward frequency bands with higher power-values, i.e., the lower frequency bands (<10 Hz).

Given the above limitations in existing analytic methods, we used the linear cue-combination theory applied in human perception studies (Ernst and Banks, 2002) to develop a method that improves the interpretability of the network's decisions. Let us consider, for example, a CNN with only 2 channels, i.e., filter maps, in the final feature tensor extracted after convolutions. Each of these filter maps preserves the spatial and/or semantic structure of the input data. Each of these filter maps acts as a cue to the network's classifier layers, denoted as *c*_1_ and *c*_2_. These cues guide the network's prediction. If we denote the desired class label as *y*_1_ and assuming *c*_1_ and *c*_2_ to be independent to each other, we can use Bayes' Theorem to write

(4) $$\begin{array}{l} P\left(y_{1}|c_{1},c_{2}\right)=\frac{P\left(c_{1},c_{2}|y_{1}\right)P\left(y_{1}\right)}{P\left(c_{1},c_{2}\right)}=\frac{P\left(c_{1}|y_{1}\right)P\left(c_{2}|y_{1}\right)P\left(y_{1}\right)}{P\left(c_{1}\right)P\left(c_{2}\right)}\\ \;=\frac{P\left(y_{1}|c_{1}\right)P\left(y_{1}|c_{2}\right)}{P\left(y_{1}\right)} \end{array}$$

If the likelihood for predicting *y*_1_ due to cue *c*_*i*_ is Gaussian with mean μ_*i*_ and variance $\sigma_{i}^{2}$, the maximum likelihood estimate (MLE) yields the combined cue, denoted by c^*^, that summarizes the important features on which the network bases its decisions. Therefore, the combined cue, c^*^, is the desired Class Activation Map (CAM).

(5) $$\begin{array}{l} c^{*}=\overset{2}{\underset{i=1}{\sum }}w_{i}\mu_{i}\text{where }w_{i}=\frac{1/\sigma_{i}^{2}}{\overset{2}{\underset{i=1}{\sum }}1/\sigma_{i}^{2}} \end{array}$$

Since the network is trained, μ_*i*_ = *c*_*i*_. To calculate the values of σ_*i*_, we used the NLL loss values. The NLL loss when a cue was removed from the network provided an estimate of the σ associated with that specific cue, as shown in Equation (6).

(6) $$\begin{array}{l} \;\epsilon =-logP\left(y_{1}|c_{1},c_{2}\right)\\ \;=-logP\left(y_{1}|c_{1}\right)-logP\left(y_{1}|c_{2}\right)+logP\left(y_{1}\right)[\text{From eq}4]\\ \;\epsilon_{1}=\epsilon {|}_{c_{1}=0}=-logP\left(y_{1}|c_{1}=0\right)-logP\left(y_{1}|c_{2}\right)+logP\left(y_{1}\right)\\ \;\epsilon_{1}-\epsilon =logP\left(y_{1}|c_{1}\right)-logP\left(y_{1}|c_{1}=0\right)\\ \;=\frac{\mu_{1}^{2}}{2\sigma_{1}^{2}}\\ \text{Therefore},\frac{1}{\sigma_{1}^{2}}=\frac{2\left(\epsilon_{1}-\epsilon \right)}{\mu_{1}^{2}} \end{array}$$

σ_*i*_ is estimated over the entire dataset as shown in Equation (7).

(7) $$\begin{array}{l} \frac{1}{\sigma_{i}^{2}}=\overset{m}{\underset{j=1}{\sum }}\frac{2{\left[\left(\epsilon_{i}-\epsilon \right)\right]}^{\left(j\right)}}{{\left[\mu_{i}^{2}\right]}^{\left(j\right)}} \end{array}$$

**Figure 3:**
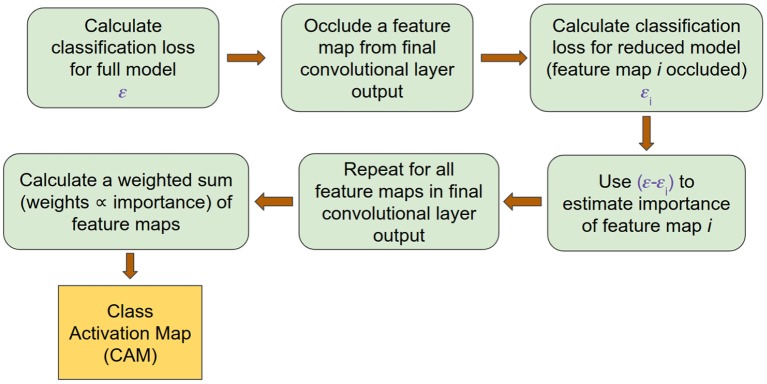
ccCAM generation pipeline outlining the procedure for obtaining CAMs from the trained deep network.

Using the estimated σ_*i*_, the CAM corresponding to the correct class for each input was obtained. Since in the present case μ_*i*_ corresponded to a 2D matrix, the denominator in Equation (7) was replaced by the mean-squared value of the corresponding matrix. A summary of the process of generating a CAM is outlined in Figure 3. The resulting CAM was a 2D matrix with each row corresponding to an electrode and each column corresponding to a frequency band. To obtain the contribution of each frequency band in determining the correct class, we averaged the CAM along the row dimension to yield a vector corresponding to the importance of each frequency band power across the whole brain. This vector was generated at each timepoint t, depicting the importance values of each frequency band at timepoint t. Since the subject was exerting a fixed force during the entire time period of the EEG segment that was considered, we expected important frequency bands to exhibit pronounced differences at all timepoints. Consequently, we considered a frequency band to be reliably modulated by exercise only if the corresponding CAM value was high for all timepoints. Therefore, we followed a procedure similar to the bootstrapping technique to estimate the reliability of each frequency band (Efron et al., 1996; McIntosh et al., 1996; McIntosh and Lobaugh, 2004). Specifically, we calculated the bootstrapped ratio (BSR) of the CAM values for each band and subject by dividing the mean of CAM values across time by its standard deviation. The obtained BSR values were subsequently group-averaged to extract frequency bands that contained features characteristic to each group (CON and EXE).

### 3.2. Topographical Maps

Topographical maps were created using the frequency bands obtained from the ccCAM corresponding to the TF-maps following the same procedure as described in Bashivan et al. (2015). Specifically, the 3D position of the electrodes on the EEG cap was projected to a 2D space to construct a 64 × 64 image. The image intensities corresponded to the respective electrode's spectral power within the frequency band of interest at time point *t*. Since this procedure yielded a sparse matrix, cubic interpolation was used to obtain a continuous image depicting the distribution of activity within each frequency band over the entire scalp. A total of three such matrices were concatenated to form a 3 × 64 × 64 tensor corresponding to activity maps at three consecutive timepoints (say *t*, *t* + 1 and *t* + 2, respectively). The entire data tensor for a given subject was created by taking non-overlapping time windows. Hence, the total number of tensors for each subject was equal to 2,500.

Similar to the analysis of TF-maps, we trained a CNN-based network to classify each data tensor into the CON and EXE groups. Since the inputs were 2D image tensors, we used 2D convolutional filters in the Base CNN (see Tables S4–S6 for more details). Following training, ccCAM was applied to obtain CAMs for each subject at each time instant during task execution.

### 3.3. Statistical Analysis for TF Curves and Topographical Maps

To estimate the statistically significant frequency bands in the resultant ccCAM maps for the two groups, we employed one-way ANOVA on a point-by-point basis. This yielded a series of *p*-values, each corresponding to a frequency bin in the ccCAM map. Since these tests were not mutually independent, we chose the Simes method (Simes, 1986; Sarkar and Chang, 1997) for correcting the obtained *p*-values as done in Loizides et al. (2015). This method was better suited to our problem compared to the standard Bonferroni correction, as several highly correlated statistics were involved. The method is based on the ordered *p*-values in a sliding window of length *L* with the *p*-value we want to correct being in the center (*L* = 2*w* + 1). The corrected *p*-value is given by

(8) $$\begin{array}{l} p_{j}^{\prime }=\underset{1\leq i\leq L}{\min}\frac{Lp_{\left(i\right)}}{i} \end{array}$$

Given the employed convolutional kernel sizes for the TF and topographical maps, we chose the corresponding window lengths for the Simes method to be 5 for TF maps and 3 × 3 for the topographical maps. All points with corrected *p*-values less than 0.05 were considered to be significant.

## 4. Results

The results presented here illustrate the differences between the baseline and 90 min post-exercise/rest datasets. The network architecture details for each type of data (TF and Topographical) map are presented in the Supplementary Material, along with details regarding the chosen hyperparameters.

### 4.1. Comparison to Alternative EEG Decoding Methods

We compared the performance of the proposed CNN to alternative machine learning methods on a leave-two-out cross-validation strategy by using 23 subjects for training the model and 2 subjects (1 from CON and 1 from EXE) for validating the model performance on unseen data. Initially, we compared the proposed CNN architectures to random forests (RF) (Breiman, 2001) and support vector machines (SVM) (Steinwart and Christmann, 2008) given the same input data. The proposed CNN with a subject adversary component clearly outperformed RF and SVM (Table 1). A feature importance analysis showed that RF was unable to account for the pink noise characteristics of EEG in the frequency domain (Dumermuth and Molinari, 1987) and therefore assigned more importance to lower frequencies. To control for this, the data were normalized by multiplying power in each frequency bin by the frequency value itself before being fed to the RF but this did not yield significant improvement.

Subsequently, the data were binned into pre-specified frequency bins, as done widely in EEG decoding techniques (Bashashati et al., 2007), and used as input in a RF. Although this improved the RF prediction performance, the resulting RF prediction accuracy was lower than the obtained CNN prediction accuracy (Table 1). Interestingly, the RF prediction accuracy was very similar to an adversary-less CNN. We also compared the performance using the common spatial pattern technique, which is widely used in EEG decoding for brain-computer interfaces (Ang et al., 2008). However, the prediction accuracy was far below the CNN prediction accuracy. Overall, these results imply that the CNN architecture with subject adversary was able to generalize better across subjects compared to the alternative, widely used EEG decoding methods. The CNN leave-two-out prediction accuracy was equal to 74.85%. The summary of the results is presented in Table 1.

To ensure a fair comparison among the different considered methods, we fixed the random seed that determined the subjects in the train-validation split for the 10 folds across all methods. The random seed was also responsible for the network initialization. By fixing the random seed, we initialized the deep network in the same state for each fold. This ensured a fairer comparison while doing the ablation experiments, where we removed certain components of the network and evaluated its performance (experiments without subject adversary or baseline normalization architecture). The average accuracy along with the standard error in accuracy for each method is shown in Table 1. To ensure reproducibility of the results, we have included the list of subjects in the validation set for each fold and the Github repository of our code base in the Supplementary Material.

The cohort size of our dataset was relatively small compared to other EEG decoding studies using deep learning. In lieu of this problem, we were unable to keep a separate test set in our analysis as this would imply having fewer subjects in the training set. Furthermore, in our experiments, we observed that there was a significant decrease in the validation accuracy when 20 subjects were kept in the training set. Instead, we used a random labeling strategy to perform a safety check against selection bias. When the labels were shuffled at the time-point level, the network was unable to converge to a solution and therefore yielded accuracy around 50% (chance prediction level). We believe that this occurred because consecutive time-points are highly correlated. Specifically, having different labels for highly correlated training data restricts the network from learning a suitable decision boundary. Therefore, we shuffled the labels at the subject level. We assigned any random combination of 13 subjects to be in the CON group and the rest in the EXE group. Following this analysis, the network trained and converged to a decision boundary but yielded a prediction accuracy of 62.57% at the time-point level. We have added these results to Table 1. The results of these experiments suggest that the accuracy achieved by our proposed CNN is not caused by a selection bias. Instead, the network recognizes the difference in EEG activity between the control and exercise groups. We believe that adding more subjects would further improve the model's performance, and thereby increase the reliability of the results. However, collecting more data or evaluating the model on other EEG datasets is beyond the scope of the current work.

### 4.2. Time-Frequency Maps

We observed that the features extracted by the Base CNN, without any subject prediction regularizer, were able to perfectly identify the subject corresponding from the obtained time-frequency patterns. As the subject discriminator regularization was given more weight by increasing λ, the Base CNN learned to extract features that were agnostic to the originating subject. However, for very high λ values, the extracted features could not be used to discriminate the EXE and CON groups, suggesting that the Base CNN was unable to learn any discriminative feature. The loss values obtained post-training for four different values of λ are shown in Table 2. The choice of an optimal value for λ depends on two factors—group prediction accuracy and subject prediction accuracy. To identify subject-invariant features, we sought for a value of λ that achieved good group prediction accuracy and poor subject prediction accuracy (i.e., a good tradeoff between the two prediction accuracies). According to this procedure, the model corresponding to λ = 13 was used for the ccCAM generation. The average loss over a batch for subject prediction was around 2.6 (Table 2), which roughly predicted the correct subject with probability of $\frac{1}{13}$ (Since $-log\left(\frac{1}{13}\right)\approx 2.6$). The group prediction accuracy was 99.984% (99.969% for CON and 100% for EXE) when evaluated using a train-test split strategy following 80-20 split of all timepoints from all subjects. Since we aimed to obtain a model that best explained the recorded EEG data, we focused on timepoint-level accuracy, instead of subject-level accuracy as before (Table 1). Therefore, we trained on 80% of timepoints from all subjects and validated the model performance on 20% unseen timepoints from all subjects. For λ = 13, the extracted features achieved excellent group prediction, while all subjects in the group were predicted with roughly equal probability (CON and EXE consisted of 13 and 12 subjects, respectively).

**Table 2:** Variation of Loss values with λ after training network on TF maps.

**λ**	**Group prediction loss (NLL)**	**Subject prediction loss (NLL)**	**KL divergence loss from Uniform distribution**
0	≈ 0	≈ 0	≈ 0.3
10	≈ 0.1	≈ 1.5	≈ 0.07
13	≈ 0.4	≈ 2.6	≈ 0.004
15	≈ 0.68	≈ 3.2	≈ 0.0002

**Figure 4:**
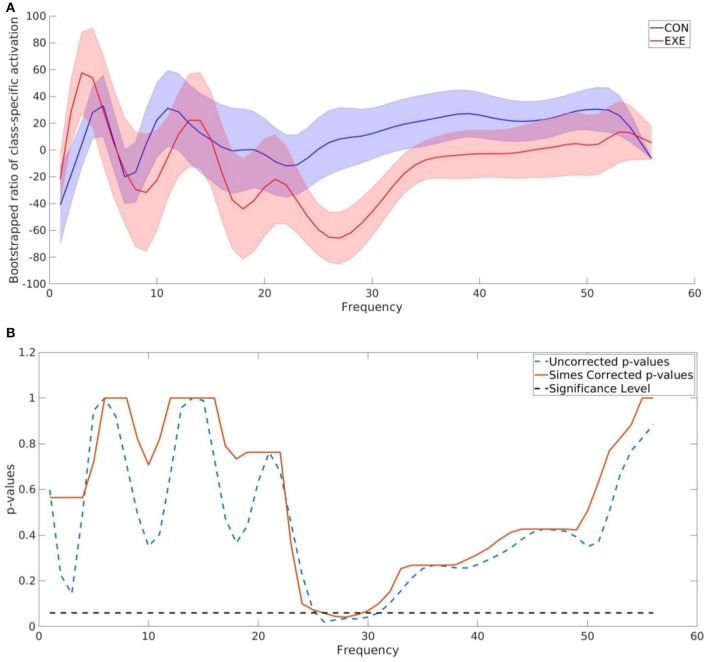
TF map ccCAM averaged over electrodes and subjects showing discriminative frequencies. **(A)** Bootstrap ratio of TF map ccCAM averaged over electrodes and subjects showing discriminative frequencies. Bootstrapping is done using ccCAM values obtained for each timepoint to establish the reliability of the ccCAM values during the task period. The two groups exhibited different BSRs within the range 23–33 Hz. **(B)** Uncorrected and Simes corrected *p*-values corresponding to the difference between the bootstrapped ratio (BSR) values of the ccCAM TF maps obtained in CON aUncorrected and Simes correctedhe BSR values between 27 and 29 Hz was found to be statistically significant (*p* < 0.05).

**Figure 5:**
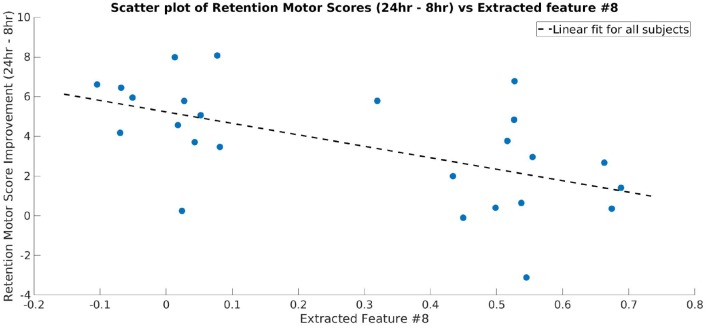
Scatter plot showing the motor skill retention improvement and a representative feature extracted by the final layer of CNN (Layer 3 of TopNN) from TF maps. The dotted line shows a linear fit between the two variables. The extracted feature is strongly correlated to the motor skill retention (correlation coefficient = −0.57).

As one of the main goals of this study was to identify the frequency bands that contained significant discriminative information between the CON and EXE groups, we calculated the BSR of the CAM values for each frequency bin as described above. The difference obtained using ccCAM BSRs is shown in Figure 4. The bold lines denote the group-mean and the shaded regions span 1 standard error over all subjects in the group. The two plots are significantly different within the band 23–33 Hz. A statistical significance analysis between the two curves with Simes correction revealed that the band 27–29 Hz lies below the significance threshold of 0.05. The uncorrected and corrected *p*-values for each frequency bin are shown in Figure 4. The aforementioned band lies within the beta-band and agrees with findings in Dal Maso et al. (2018), where beta-band desynchronization was found to be significantly modulated by exercise. Note that Figure 4 corresponds to the differences between the 90 min and baseline EEG recordings. In addition, the features extracted by the CNN were found to be strongly correlated to the motor skill retention improvement between the 8 and 24 h session (Figure 5).

**Figure 6:**
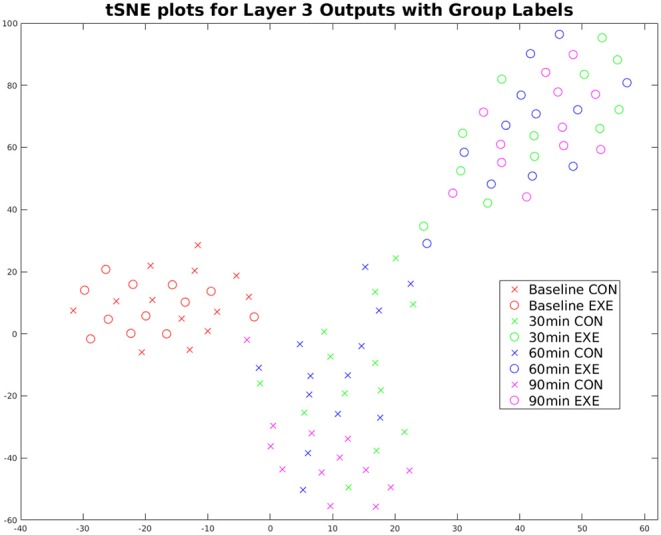
t-SNE plot of Top NN Layer 3 outputs (time-averaged) for the TF maps for all subjects and sessions. The EXE subjects (circles) move further away from baseline with time compared to the CON group (crosses), indicating exercise-induced changes on the underlying electrophysiological signals. Also, the feature trajectories across time are very different for the two groups starting from the 30 min session, which indicates that exercise-induced changes surface right after the session of acute exercise.

The CNN was trained to classify the EXE and CON groups given the baseline EEG and 90 min post-exercise EEG recordings. To observe how the discriminative features evolved over time (30 and 60 min post-exercise), we used the t-SNE algorithm (Maaten and Hinton, 2008) to obtain a lower dimensional (2D) representation of the feature vectors extracted by the trained CNN for all sessions and subjects. Figure 6 shows the corresponding plot with each point representing one session from one subject, color-coded with respect to session and group. The differences between the features extracted from the 90 min post-exercise and baseline sessions were more pronounced as compared to the EXE group as compared to the CON group. Also, the difference between the two groups becomes prominent from the 30 min post-exercise session itself, with the feature trajectories being considerably different for the two groups.

### 4.3. Topographical Maps

**Figure 7:**
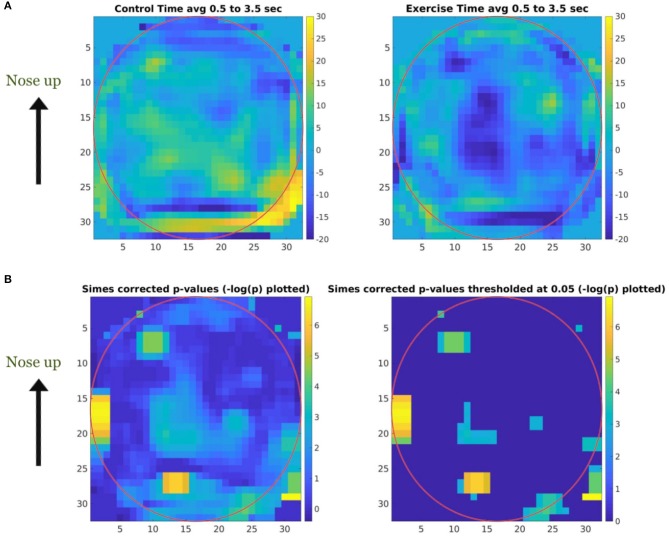
Topographical map ccCAM averaged over subjects showing brain areas with discriminative activity. **(A)** Bootstrapped ratio of the topographical map of ccCAM values averaged over subjects in the CON and EXE groups showing regions with difference in activity before and 90 min after rest/exercise. Bootstrapping was performed using ccCAM values obtained for each timepoint to establish the reliability of the ccCAM values during the task period. **(B)** Topographical maps of significantly different ccCAM bootstrapped ratio values between the CON and EXE groups. Instead of *p*-values, −*log(p)* is color coded to delineate significant regions (yellow) more clearly. Non-significant regions are shown in blue. Significantly different activity was observed over the sensorimotor, occipital, and frontal areas.

**Figure 8:**
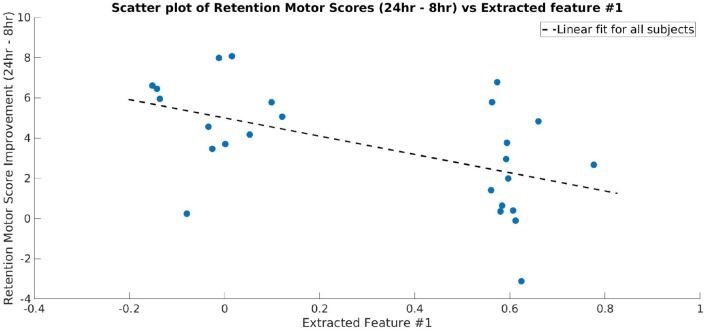
Scatter plot showing the motor skill retention improvement and a representative feature extracted by the final layer of CNN (Layer 3 of TopNN) from topographical maps. The dotted line shows a linear fit between the two variables. The extracted feature is strongly correlated to the motor skill retention (correlation coefficient = −0.53).

Topographical maps were created to study the distribution of the activity within the 23–33 Hz frequency band across the cortex. Since we used a convolutional filter of size 5 in the BaseCNN for TF-maps, we selected the wider 23–33 Hz instead of the finer 27–29 Hz band, and projected this band-limited activity on the cortex to obtain topographical maps. After training a network to classify the CON and EXE groups from the resulting topographical maps, a classification accuracy of 98.70% (98.94% for CON and 98.43% for EXE) was obtained for λ = 5. Generating ccCAMs for the topographical maps revealed brain areas where the activity was notably different between the CON and EXE groups. The BSR values were obtained to estimate the reliability as in the case of TF maps and the results are shown in Figure 7. Statistical analysis to highlight areas with significantly different activity within the 23–33 Hz band is shown in Figure 7. Notable areas that exhibited significant differences include the contralateral and ipsilateral sensorimotor areas, the contralateral prefrontal area and the occipital areas. The observed differences in occipital areas are not surprising as the occipital cortex is primarily responsible for visual information processing and the task under consideration is a visuomotor task. Therefore, these differences could also highlight a change in functional connectivity between the sensorimotor and occipital cortices. Findings in sensorimotor and prefrontal areas are in strong agreement with those reported in Dal Maso et al. and therefore suggest that the proposed method was able to identify the modulatory effect of exercise (Dal Maso et al., 2018). In addition, the topographical features extracted by the CNN were strongly correlated to the motor skill retention improvement between the 8 hr and 24 hr sessions (Figure 8), indicating a possible association of these features to the observed motor learning improvements in the EXE group (Dal Maso et al., 2018).

## 5. Discussion

In the present work, we used a novel DL approach to investigate the effects of acute cardiovascular exercise on brain activity during the early stages of motor memory consolidation while subjects performed isometric handgrips. The proposed methodological approach does not require the specification of *a priori* features and included a novel feature visualization technique to highlight the neurophysiological patterns modulated by exercise. Our approach addressed existing caveats related to the application of DL architectures, such as CNNs, to EEG data recorded from a relatively small number of subjects by means of three novel contributions.

1. We used two parallel feature extraction streams to identify informative features from EEG data before and after a session of cardiovascular exercise and subsequently characterize the modulatory effect on these baseline-corrected features rather than on the raw EEG data;
2. We incorporated a subject prediction adversary component in the network architecture to learn subject-invariant, group-related features instead of subject-specific features;
3. We developed a novel feature visualization method, termed cue-combination for Class Activation Map (ccCAM).

Previous studies using DL in the context of structural and functional neuroimaging (Plis et al., 2014; Bashivan et al., 2015; Thodoroff et al., 2016; Schirrmeister et al., 2017b) have been primarily restricted to classification tasks and have relied on a large cohort of subjects for training. The interpretation of the results obtained from these studies and their usefulness for investigating neuroscientific questions has been less straightforward, primarily due to the difficulty associated with the visualization and interpretation of the feature space learned by the employed DL architectures. Schirrmeister et al. proposed a feature validation approach to understand which *a priori* selected features were given importance by a CNN that was trained to decode imagined or executed tasks from raw EEG (Schirrmeister et al., 2017b). Specifically, their method relied on calculating correlations between the output of each unit or layer of the network and EEG power in specific frequency bands. This allowed them to verify if the network used the power in *a priori* decided frequency bands for its predictions. Additional studies involving CNNs for EEG decoding have extended the deep dream algorithm (Mahendran and Vedaldi, 2015, 2016) for identifying discriminative features in EEG time segments (Putten et al., 2018). However, this algorithm was found to be sensitive to the scale of features and therefore may not be applicable to a diverse range of neuroimaging studies. The proposed ccCAM methodology is a feature discovery technique that is less sensitive to the feature scale and thereby allows extending the applicability of CNNs beyond classification tasks. Furthermore, our study introduced a novel adversary component to prevent the CNN from exclusively learning subject-specific features. This allowed the DL pipeline to learn group-specific features from the EEG data and therefore improved the generalizability of the CNN from a limited cohort size. Overall, to the best of our knowledge, our study is the first to investigate neuroscience-based questions, specifically related to motor learning, from a limited cohort size using a DL approach.

Our analysis using CNNs outperformed alternative machine learning and EEG decoding methods with regards to differentiating between previously unseen EXE and CON subjects, thus indicating the improved generalization properties of the proposed method. The proposed ccCAM method was able to identify a finer frequency band (27–29 Hz) that was modulated by exercise, as opposed to the entire beta band reported in Dal Maso et al. (2018), without using any prior knowledge. Applying ccCAM to the topographical patterns of the electrophysiological activity between 23 and 33 Hz revealed the specific brain areas (the contralateral and ipsilateral sensorimotor areas, contralateral prefrontal area and occipital areas) that were mostly impacted by exercise in agreement to previous studies (Dal Maso et al., 2018). Correlation analysis between the extracted features and the motor learning scores yielded further evidence that these features are plausible neurophysiological substrates underlying the positive effects of exercise on motor learning.

### 5.1. Comparison to Alternative EEG Decoding Methods

A comparison of the proposed DL pipeline to alternative machine learning methods, such as RF showed that it yielded improved generalization performance to unseen subjects (Table 1). In turn, this suggests that the DL pipeline was able to learn features that were specific to the exercise intervention. Specifically, the performance of RF was close to that of CNNs without an adversary. This suggests the importance of the adversary component in learning subject-invariant, group-specific features. A feature importance analysis for the RF models indicated that higher importance was assigned to features corresponding to lower frequency bands, which are more generally associated with cognitive behaviors (Jensen and Tesche, 2002). Therefore, the absence of an adversary component possibly caused the examined machine learning models to assign higher importance scores to these subject-specific features, which were more likely reflective of the cognitive state of the subject while performing the task. In turn, this may explain their more limited generalizability to unseen subjects.

The CNN leave-two-out prediction accuracy was equal to 74.85%, which was lower compared to the accuracy achieved by CNNs in computer vision or motor imagery EEG datasets (Schirrmeister et al., 2017b). We believe that the main reason for this is the number of subjects and that adding more subjects would yield improved accuracy. Furthermore, we did not compare the classification accuracy of CNN architectures that have been popularly used for EEG decoding because those networks were designed specifically for raw time-series data (Schirrmeister et al., 2017b; Lawhern et al., 2018). The main focus of our work was to train a CNN that could discriminate the two groups from their spectral EEG patterns before and after an exercise session.

### 5.2. Time-Frequency Maps

The ccCAM approach was able to identify the frequency bands that were significantly different in terms of ERD patterns between the EXE and CON groups (Figure 4). Specifically, the frequency band between 27–29 Hz, which is a subset of the wider beta band (15–29 Hz) typically used in previous studies related to motor activity-associated ERD, yielded the most significant differences. This finding is in agreement with Dal Maso et al. (2018) and implies that decreased neural excitability was needed to perform the handgrip task after exercise. The *p*-value calculated from the obtained time-frequency data within the 27–29 Hz frequency band was equal to **0.0044**. This suggests that if the band of interest in Dal Maso et al. (2018) had been chosen to be 27–29 Hz, instead of the entire beta-band (15–29 Hz), similar statistically significant results would have been obtained. The standard error values in the ccCAM reliability plots (Figure 4) indicate that the frequency band modulated is variable among subjects. Overall, the proposed DL-based analysis was able to identify a narrower frequency band modulated by exercise without using any prior knowledge. This finding has particular importance for the development of targeted interventions, such as non-invasive stimulation protocols, whereby it is desirable to modulate the frequency band of interest without interfering with other frequencies.

The trajectory of the extracted features was significantly different for the CON and EXE groups (Figure 6), indicating that the effects of exercise were observable during the consolidation period. However, it cannot be concluded from these results whether these changes are short- or long-term. Measuring the brain activity of the subjects at a later timepoint (e.g., 24 h later) may reveal further insights about the evolutionary patterns of exercise-induced changes during the retention period.

An important goal of the present study was to identify whether there was an association between the identified brain features and the learning scores of each subject. The features indeed were found to be strongly correlated to the motor learning improvement assessed by the difference between the skill retention scores at 8 and 24 h after motor practice (Figure 5). As reported by Dal Maso et al. (2018), these effects probably indicate the effect of sleep on motor memory consolidation (King et al., 2017). This strongly suggests that the features extracted by the CNN corresponded to exercise-induced changes while carrying predictive information about the subject's motor skill retention abilities following a period of sleep.

### 5.3. Topographical Maps

The distribution of the discriminatory 23–33 Hz band power across the brain was found to be localized instead of being widespread across the brain (Figure 7), suggesting that activity in specific brain regions was modulated by exercise. Notable areas with differential activity included the contralateral and ipsilateral sensorimotor areas, contralateral prefrontal area and occipital areas. These results are in strong agreement with the inferences drawn in Dal Maso et al. (2018). Interestingly, the analysis pipeline employed in the present work uncovered the areas that were differentially activated in the two groups, whereas the same inferences required the use of 3 different metrics in the standard analysis performed by Dal Maso et al. Specifically, in Dal Maso et al. (2018) the differences in ERD were observed in the sensorimotor areas, differential functional connectivity was observed among the sensorimotor and occipital areas and the ERD in contralateral prefrontal area electrode activity was shown to have strong correlations to motor score. In comparison, using only the ERD values from the baseline and post-exercise sessions, the proposed DL approach was able to identify the brain regions modulated by exercise without any *a priori* knowledge.

Similar to what was reported in Dal Maso et al., we found reduced ERD in the sensorimotor areas as well as the contralateral prefrontal area, suggesting a more efficient use of neural substrates involved in motor memory consolidation (van Wijk et al., 2012). Consequently, these results indicated that after exercise, reduced neural excitability in these areas was required to perform the fixed force handgrip task. As discussed in Dal Maso et al., the observed decrease in ERD could also be indicative of a reduction in gamma-aminobutyric acid (GABA) inhibitory activity due to exercise (Singh and Staines, 2015). Given that the task was a visuomotor one, we also expected changes in the visual areas. Dal Maso et al. reported these changes by using functional connectivity analysis within the beta band. However, using a finer band revealed ERD changes in these areas and these could be indicative of changes in visual attention activity and perception. The range of beta band activity commonly used in visual studies is 15–25 Hz and is thought to be a carrier for visual attention, whereas gamma band (30–60 Hz) activity is thought to be responsible for visual synchronization and perception (Wróbel, 2000). Interestingly, the range of frequencies uncovered by our analysis included high beta activity and low gamma activity. Therefore, the proposed DL pipeline highlighted a modulation of these properties by a session of exercise. As we used a visuomotor task, it is difficult to delineate the modulation effects in visual areas from motor areas and thereby draw conclusive inferences about findings in visual areas. The significantly different activity in the prefrontal area aligns well with prior literature demonstrating the role of the dorsolateral prefrontal cortex in motor memory consolidation (Galea et al., 2010). Therefore, it is plausible that acute cardiovascular exercise promotes the efficient distribution of neural resources in the prefrontal area (Dietrich, 2006), thus reducing neural demands of cognitive processes that underlie the consolidation of motor memory. Interestingly, the CNN-extracted topographical features are well correlated to motor learning improvement measured after a period of sleep (Figure 8), i.e., once the memory has been well consolidated (Roig et al., 2016). Taken together, the identified features are indicative of the resultant consolidated memory and not necessarily behavior during the memory consolidation period.

The presented results provide observational evidence for the extracted features to be considered as candidate neurophysiological substrates underlying motor memory consolidation. As argued by Tonegawa et al. (2015), observational studies demonstrate a correlation between specific neural activity and behavior and therefore act as preliminary evidence for establishing causality. Future studies need to target loss-of-function and gain-of-function experiments to establish a causation link between neurophysiological mechanisms and motor memory consolidation. The proposed method achieved the identification of more specific, narrower frequency band activity modulated by cardiovascular exercise that was unique to each subject. To establish a stronger causal link between frequency band-related ERD and the positive effects of exercise, future studies could modulate activity in these subject-specific bands by non-invasive electrical stimulation techniques and subsequently assess motor learning behavior. Our analysis pipeline also has clinical relevance as it could potentially be used to estimate the efficacy of rehabilitation strategies for individual subjects, e.g., stroke patients. Neurophysiological features modulated by the intervention could be extracted using the presented DL pipeline and subsequently used to obtain improvements in performance. Therefore, the proposed methodology yields significant potential for designing of patient-specific neurorehabilitation therapies that can significantly improve upon a “one-size-fits-all” approach.

### 5.4. Limitations

A major limitation of the CNN-based approach lies in computational demands. CNNs require more time and specialized hardware, namely GPUs, to train. In our case, CNNs were trained on a GeForce GTX 960 and required around 7 h to train. The training demands and consequently required time would increase with more subjects. Therefore, the operational cost of using the presented methodology is expected to be higher as compared to alternative state-of-art methods.

The sample size used in this work was limited to 25 subjects. Due to this, we were unable to keep a separate test set while evaluating the network's performance. We observed that there was a significant decrease in the validation accuracy when 20 subjects were kept in the training set. Therefore, we persisted with a leave-two-subjects-out cross-validation strategy and were unable to hold out a separate test set. We believe that adding more subjects would further improve the model's performance and test it on a separate test set, thereby increasing the overall reliability of the results. However, collecting more data or evaluating the model on other EEG datasets is beyond the scope of the current work.

## 6. Conclusion

The present work introduces a deep learning architecture for the analysis of EEG data and shows promising results in terms of discriminating the effects of an acute bout of high-intensity exercise/rest in close temporal proximity to performing a motor learning task on the brain activity of participants. The proposed approach outperformed alternative machine learning methods in terms of the classification of CON and EXE subjects. Importantly, it also enabled us to visualize the features learned by deep networks such as CNNs, which may in turn yield better feature interpretation. The results are in general agreement with those reported in a previous study using standard statistical analysis using *a priori* selected features on the same dataset (Dal Maso et al., 2018), with our analysis revealing a narrower, more specific frequency band associated with exercise-induced changes. In addition, our method revealed localized regions of the differential activity. Therefore, our approach demonstrates the feasibility of identifying subtle discriminative features in a completely data-driven manner using deep learning.

The proposed method is not restricted to the EEG modality and dataset described here. Hence, it paves the way for applying similar methods to other neuroimaging datasets of differing cohort sizes. This, in turn, yields promise for using deep learning as a tool toward the identification of neurophysiological changes associated with a variety of neurological disorders and ultimately lead to the design of optimized and individualized intervention strategies.

## Data Availability Statement

The source-code used to perform the analysis is available on GitHub (https://github.com/BioSigSystLab/EEG-exercise-DeepLearning) and the accompanying data used in the study will be made available upon request.

## Ethics Statement

The studies involving human participants were reviewed and approved by CRIR Research ethics board (CRIR-1134-0116). The patients/participants provided their written informed consent to participate in this study.

## Author Contributions

AG did the analysis, implemented the CNN training, and ccCAM technique. FD was involved in the preprocessing of data and data collection. MR designed the experiment protocol and supervised data collection. GM supervised the design of the analysis pipeline and was involved in the interpretation of results. M-HB designed the experiment protocol, supervised data collection, and supervised the analysis pipeline as well as interpretation of results.

### Conflict of Interest

The authors declare that the research was conducted in the absence of any commercial or financial relationships that could be construed as a potential conflict of interest.
