# Supplementary material for: Unfolding the Effects of Acute Cardiovascular Exercise on Neural Correlates of Motor Learning Using Convolutional Neural Networks
Source: Front Neurosci. 2019 Nov 14;13:1215. doi: 10.3389/fnins.2019.01215 (PMC6868001; doi:10.3389/fnins.2019.01215)
Supplement: Supplementary file 1 [file Data_Sheet_1.pdf]

Unfolding the effects of acute cardiovascular exercise on neural correlates of motor learning using Convolutional Neural Networks

Arna Ghosh
Integrated Program in Neuroscience
McGill University
Montréal, QC H3A 0G4. Canada
arna.ghosh@mail.mcgill.ca

Fabien Dal Maso
École de kinésiologie et des sciences de l'activité physique
Université de Montréal
Montréal, QC H3T 1J4. Canada
fabien.dal.maso@umontreal.ca

Marc Roig
School of Physical and Occupational Therapy
McGill University
Montréal, QC H3A 0G4. Canada.
marc.roigpull@mcgill.ca

Georgios D Mitsis
Department of Bioengineering
McGill University
Montréal, QC H3A 0G4. Canada.
georgios.mitsis@mcgill.ca

Marie-Hélène Boudrias
School of Physical and Occupational Therapy
McGill University
Montréal, QC H3A 0G4. Canada.
mh.boudrias@mcgill.ca

Supplementary Material

The code base is publicly available on Github - Link

1 Network Architecture

Notation:- *Conv* denotes the 2D Spatial Convolutional layer. *ReLU* denotes the Rectified Linear Unit Layer that adds non-linearity to the network. *MaxPool* denotes the 2D Spatial Max Pooling layer. *FullyConn* denotes a Fully Connected layer, also known as the linear layer of the network.

1.1 TF maps

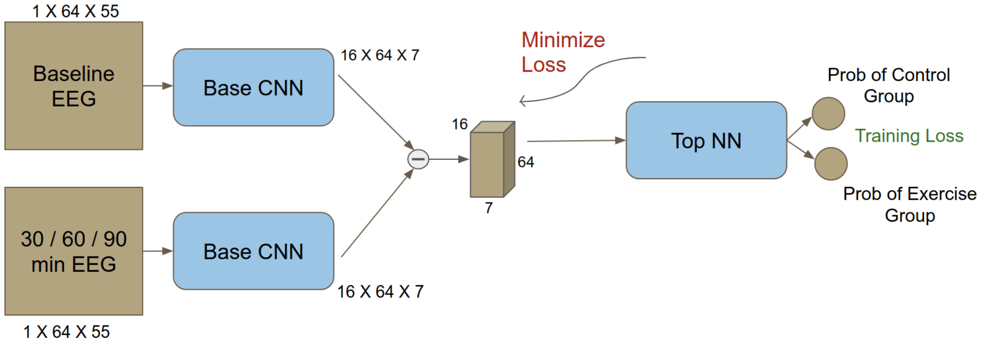

(a) Basic Architecture without adversary

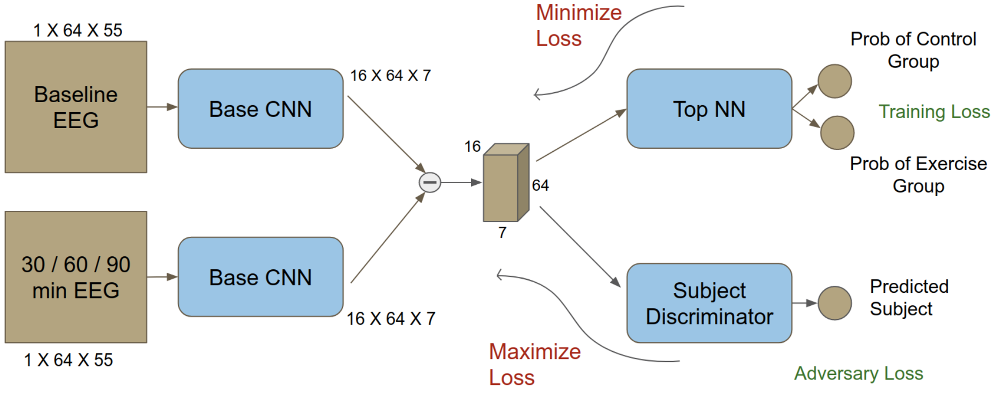

(b) Modified Architecture with adversary to avoid subject discrimination

Figure S1: Deep Network Architecture. The initial choice of architecture (without any adversary) gives good subject prediction accuracy from features extracted by the Base CNN. Therefore, a subject discriminator of roughly the same model capacity as the Top NN is added. The subject discrimination acts as a regularizer while training and avoids the Base CNN from learning subject specific features.

Layer	Type	Maps and Neurons	Filter Size
0	Input	1M $\times$ 64 $\times$ 55N	-
1	Conv	6M $\times$ 64 $\times$ 28N	1$\times$5
2	ReLU	6M $\times$ 64 $\times$ 28N	-
3	MaxPool	6M $\times$ 64 $\times$ 14N	1$\times$2
4	Conv	16M $\times$ 64 $\times$ 14N	1$\times$5
5	ReLU	16M $\times$ 64 $\times$ 14N	-
6	MaxPool	16M $\times$ 64 $\times$ 7N	1$\times$2

Table S1: Network architecture used for EEG feature extraction network (Base CNN). The output of the network is a tensor of dimensions $16 \times 64 \times 7$.

Layer	Type	Maps and Neurons	Filter Size
0	Input	16M $\times$ 64 $\times$ 7N	-
1	Flatten	7168N	-
2	Dropout (p=0.5)	-	-
3	FullyConn	8N	1$\times$1
4	ReLU	8N	-
5	FullyConn	2N	1$\times$1

Table S2: Network architecture used for group discrimination network (Top NN). The output of the network is a vector of dimension 2, values corresponding to the probability that the data tuple belongs to particular class.

Layer	Type	Maps and Neurons	Filter Size
0	Input	16M $\times$ 64 $\times$ 7N	-
1	Flatten	7168N	-
2	Dropout (p=0.5)	-	-
3	FullyConn	8N	1$\times$1
4	ReLU	8N	-
5	FullyConn	25N	1$\times$1

Table S3: Network architecture used for subject discrimination network (adversary). The output of the network is a vector of dimension 25, values corresponding to the probability that the data tuple belongs to particular subject.

1.2 Topographical maps

Layer	Type	Maps and Neurons	Filter Size
0	Input	3M × 64 × 64N	-
1	Conv	16M × 32 × 32N	5×5
2	ReLU	16M × 32 × 32N	-
3	MaxPool	16M × 16 × 16N	2×2
4	Conv	32M × 16 × 16N	5×5
5	ReLU	32M × 16 × 16N	-
6	MaxPool	32M × 8 × 8N	2×2
7	Conv	64M × 8 × 8N	3×3
8	ReLU	64M × 8 × 8N	-
9	MaxPool	64M × 4 × 4N	2×2

Table S4: Network architecture used for EEG feature extraction network (Base CNN). The output of the network is a tensor of dimensions $64 \times 4 \times 4$.

Layer	Type	Maps and Neurons	Filter Size
0	Input	64M × 4 × 4N	-
1	Flatten	1024N	-
2	Dropout (p=0.5)	-	-
3	FullyConn	8N	1×1
4	ReLU	8N	-
5	FullyConn	2N	1×1

Table S5: Network architecture used for group discrimination network (Top NN). The output of the network is a vector of dimension 2, values corresponding to the probability that the data tuple belongs to particular class.

Layer	Type	Maps and Neurons	Filter Size
0	Input	64M × 4 × 4N	-
1	Flatten	1024N	-
2	Dropout (p=0.5)	-	-
3	FullyConn	8N	1×1
4	ReLU	8N	-
5	FullyConn	25N	1×1

Table S6: Network architecture used for subject discrimination network (adversary). The output of the network is a vector of dimension 25, values corresponding to the probability that the data tuple belongs to particular subject.

2 Train Validation split details

Fold	CON subject	EXE subject
1	12	25
2	2	15
3	8	19
4	3	14
5	1	22
6	12	14
7	1	20
8	9	17
9	12	18
10	4	15

Table S7: List of subjects in the validation set for each fold of 10-fold cross-validation setup.

3 Training curves

3.1 Time-Frequency Maps

Hyperparameter	Value
Learning Rate	0.002
Learning Rate Decay	0.0001
Weight Decay	0.001

Table S8: List of hyperparameters used for training the networks on TF maps.

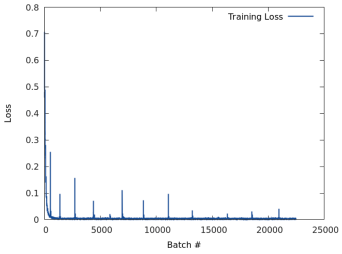

(a) Group prediction loss ($\lambda = 0$)

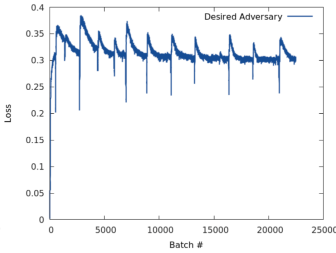

(b) KL divergence ($\lambda = 0$)

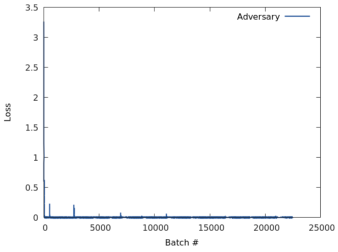

(c) Subject prediction loss ($\lambda = 0$)

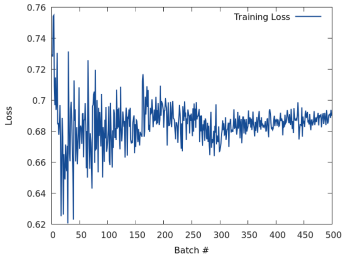

(d) Group prediction loss ($\lambda = 15$)

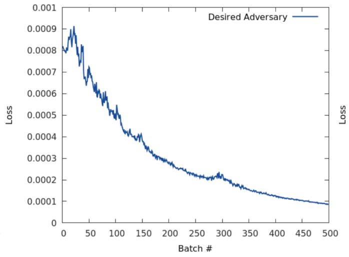

(e) KL divergence ($\lambda = 15$)

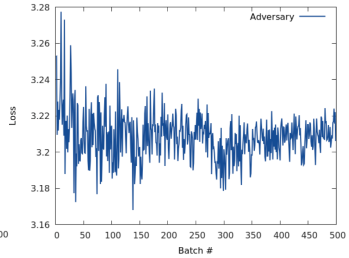

(f) Subject prediction loss ($\lambda = 15$)

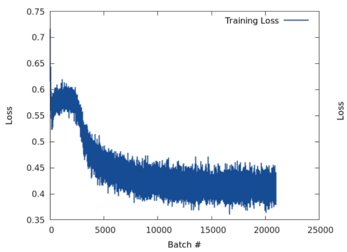

(g) Group prediction loss ($\lambda = 13$)

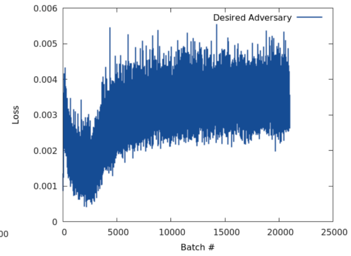

(h) KL divergence ($\lambda = 13$)

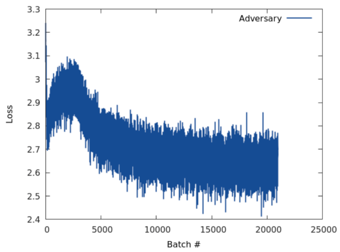

(i) Subject prediction loss ($\lambda = 13$)

Figure S2: Time-Frequency Maps Training curves for three different weight values to the subject predictor regularizer.

3.2 Topographical Maps

Hyperparameter	Value
Learning Rate	0.001
Learning Rate Decay	0.001
Weight Decay	0.03

Table S9: List of hyperparameters used for training the networks on Topographical maps.

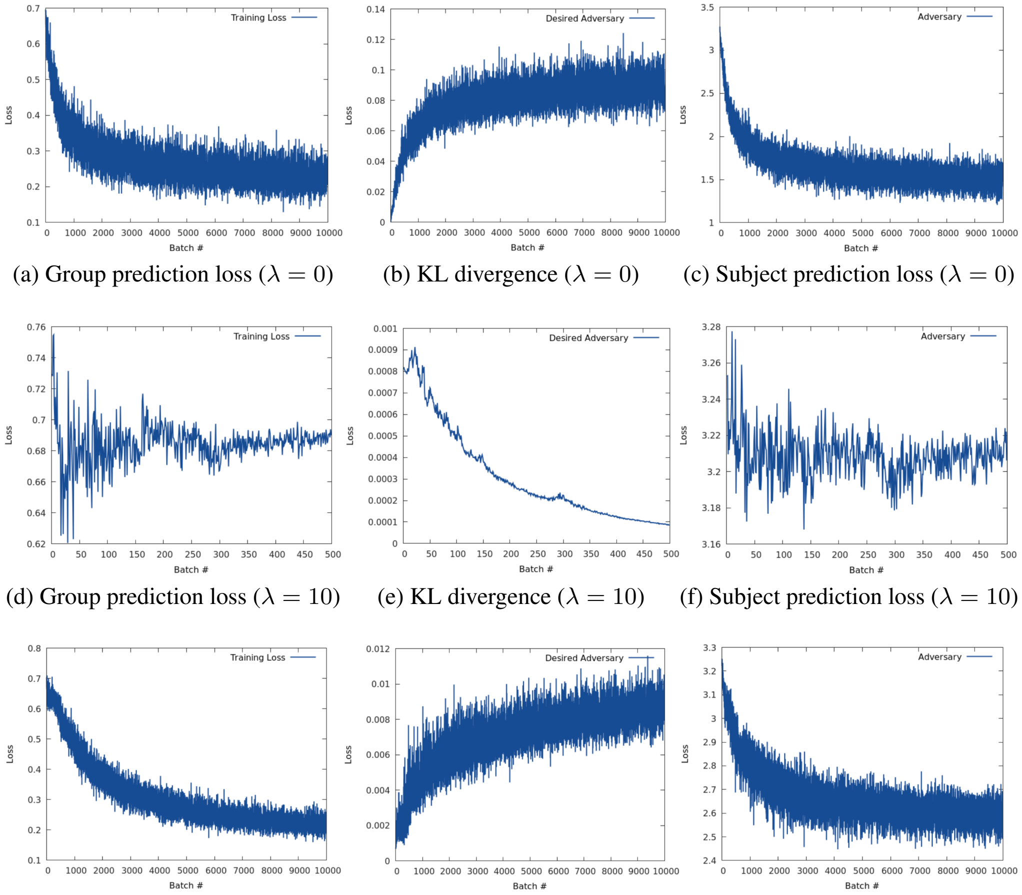

(a) Group prediction loss ($\lambda = 0$) (b) KL divergence ($\lambda = 0$) (c) Subject prediction loss ($\lambda = 0$)

(d) Group prediction loss ($\lambda = 10$) (e) KL divergence ($\lambda = 10$) (f) Subject prediction loss ($\lambda = 10$)

(g) Group prediction loss ($\lambda = 5$) (h) KL divergence ($\lambda = 5$) (i) Subject prediction loss ($\lambda = 5$)

Figure S3: Topographical Maps Training curves for three different weight values to the subject predictor regularizer.
